# Identification of LukPQ, a novel, equid-adapted leukocidin of *Staphylococcus aureus*

**DOI:** 10.1038/srep40660

**Published:** 2017-01-20

**Authors:** Gerrit Koop, Manouk Vrieling, Daniel M. L. Storisteanu, Laurence S. C. Lok, Tom Monie, Glenn van Wigcheren, Claire Raisen, Xiaoliang Ba, Nicholas Gleadall, Nazreen Hadjirin, Arjen J. Timmerman, Jaap A. Wagenaar, Heleen M. Klunder, J. Ross Fitzgerald, Ruth Zadoks, Gavin K. Paterson, Carmen Torres, Andrew S. Waller, Anette Loeffler, Igor Loncaric, Armando E. Hoet, Karin Bergström, Luisa De Martino, Constança Pomba, Hermínia de Lencastre, Karim Ben Slama, Haythem Gharsa, Emily J. Richardson, Edwin R. Chilvers, Carla de Haas, Kok van Kessel, Jos A. G. van Strijp, Ewan M. Harrison, Mark A. Holmes

**Affiliations:** 1 Department of Farm Animal Health, Faculty of Veterinary Medicine, Utrecht University, 3584 CL, Utrecht, The Netherlands; 2 Medical Microbiology, University Medical Center Utrecht, 3584 CX Utrecht, The Netherlands; 3 Department of Medicine, University of Cambridge School of Clinical Medicine, Addenbrooke’s and Papworth Hospitals, Hills Road, Cambridge CB2 0QQ, United Kingdom; 4 Medical Research Council Human Nutrition Research, Elsie Widdowson Laboratory, 120 Fulbourn Road, Cambridge CB1 9NL, United Kingdom; 5 Department of Veterinary Medicine, University of Cambridge, Cambridge CB3 0ES, United Kingdom; 6 Department of Infectious Diseases and Immunology, Faculty of Veterinary Medicine, Utrecht University, 3584 CL Utrecht, The Netherlands; 7 Central Veterinary Institute of Wageningen UR, 8200 AB Lelystad, The Netherlands; 8 The Roslin Institute, University of Edinburgh, EH25 9RG, Edinburgh, United Kingdom; 9 Moredun Research Institute, Bush Loan, Penicuik EH26 0PZ, United Kingdom; 10 Institute of Biodiversity, Animal Health and Comparative Medicine, College of Medical, Veterinary and Life Sciences, University of Glasgow, Glasgow G61 1QH, United Kingdom; 11 Royal (Dick) School of Veterinary Studies, University of Edinburgh, Easter Bush Campus, Midlothian, EH25 9RG, United Kingdom; 12 Área Bioquímica y Biología Molecular, Universidad de La Rioja, Madre de Dios 51, Logroño 26006, Spain; 13 Animal Health Trust, Lanwades Park, Kentford, Newmarket CB8 7UU, United Kingdom; 14 Department of Clinical Sciences and Services, Royal Veterinary College, Hawkshead Lane, Hatfield, North Mymms, Hertfordshire AL9 7TA, United Kingdom; 15 Institute of Microbiology, University of Veterinary Medicine Vienna, Veterinärplatz 1, 1210 Vienna, Austria; 16 Department of Veterinary Preventive Medicine, College of Veterinary Medicine, The Ohio State University, 1900 Coffey Road, Columbus, OH 43210, USA; 17 Veterinary Public Health Program, College of Public Health, The Ohio State University, 1900 Coffey Road, Columbus, OH 43210, USA; 18 Department of Animal Health and Antimicrobial Strategies, SVA, SE-751 89 Uppsala, Sweden; 19 Department of Veterinary Medicine and Animal Production, Infectious Diseases Section, University of Naples “Federico II”, 80137 Naples, Italy; 20 Interdisciplinary Centre of Research in Animal Health, Faculdade de Medicina Veterinária, Universidade de Lisboa, 1300-477 LISBOA, Portugal; 21 Laboratório de Genética Molecular, Instituto de Tecnologia Química e Biológica da Universidade Nova de Lisboa (ITQB/UNL), Oeiras, Portugal; 22 Laboratory of Microbiology and Infectious Diseases, The Rockefeller University, New York, NY10065, USA; 23 Laboratoire de Microorganismes et Biomolécules actives, Département de Biologie, Faculté de Sciences de Tunis, 2092 Tunis, Tunisia; 24 Institut Supérieur des Sciences Biologiques Appliquées de Tunis, Université de Tunis El Manar, 2092 Tunis, Tunisia; 25 Institute of Microbiology and Infection, University of Birmingham, Birmingham B15 2TT, UK; 26 Department of Medicine, University of Cambridge, Addenbrooke’s Hospital, Cambridge CB2 0QQ, UK

## Abstract

Bicomponent pore-forming leukocidins are a family of potent toxins secreted by *Staphylococcus aureus*, which target white blood cells preferentially and consist of an S- and an F-component. The S-component recognizes a receptor on the host cell, enabling high-affinity binding to the cell surface, after which the toxins form a pore that penetrates the cell lipid bilayer. Until now, six different leukocidins have been described, some of which are host and cell specific. Here, we identify and characterise a novel *S. aureus* leukocidin; LukPQ. LukPQ is encoded on a 45 kb prophage (ΦSaeq1) found in six different clonal lineages, almost exclusively in strains cultured from equids. We show that LukPQ is a potent and specific killer of equine neutrophils and identify equine-CXCRA and CXCR2 as its target receptors. Although the S-component (LukP) is highly similar to the S-component of LukED, the species specificity of LukPQ and LukED differs. By forming non-canonical toxin pairs, we identify that the F-component contributes to the observed host tropism of LukPQ, thereby challenging the current paradigm that leukocidin specificity is driven solely by the S-component.

The human and animal pathogen *Staphylococcus aureus* is capable of colonizing and infecting a broad range of host species. *S. aureus* has been shown to adapt to its hosts through acquisition of mobile genetic elements and the introduction of allelic variation through chromosomal mutations. For example, ruminant and equine *S. aureus* strains have acquired pathogenicity islands encoding host-specific variants of von Willebrand factor-binding protein^1^,^2^ and recently a single nucleotide polymorphism in the *dltB* gene was shown to make a human *S. aureus* strain capable of infecting rabbits^3^.

Leukocidins are a family of bicomponent pore-forming toxins contributing to *S. aureus* pathogenicity. Currently there are six known leukocidins of *S. aureus* (HlgAB, HlgCB, LukAB/HG, LukED, Panton-Valentine leukocidin (LukSF-PV/PVL), and LukMF’), all consisting of two subunits (an S- and an F-component) that together induce pore formation. In the current model of pore formation, the S-component first binds to a specific receptor on the cell surface, after which the F-component can associate to form octameric beta-barrel pores in the host cell membrane^4^. Both gamma-hemolysins (*hlgAB* and *hlgCB*) and *lukAB/HG* are encoded in the core genome of *S. aureus*, while *lukED* is located on a common pathogenicity island (vSaβ). In contrast, *pvl* and *lukMF’* are located on prophages^4^. While *pvl* is mostly found in *S. aureus* isolates from human origin, *lukMF*’ is almost exclusively harboured by strains from ruminant origin^5^,^6^,^7^,^8^. Corresponding with their distribution, these leukocidins display specific host tropisms, explained by the high-affinity interaction of the toxins with receptor molecules which differ between host species^9^,^10^,^11^,^12^. This leads to large differences in leukocidin activity between host species. For example, PVL has been shown to lyse neutrophils from rabbits and humans, but to have no effect on Java monkey neutrophils^13^, while LukMF´ is highly toxic to ruminant neutrophils, but not to human neutrophils^14^,^15^.

Here, we describe a novel phage-encoded member of the *S. aureus* bicomponent leukocidin family named LukPQ, which shares 91% and 80% amino-acid sequence identity with LukE and LukD respectively. We show that LukPQ is strongly associated with *S. aureus* strains isolated from *Equidae* (horses and donkeys) and, in accordance with this distribution, preferentially kills neutrophils from equine origin with a higher efficiency than its closest relative LukED. We identify the equine-CXCRA and CXCR2 as receptors for the S-component, but, in contrast to the current paradigm, we show that the observed host specificity is not solely determined by the S-component, but also in part by the F-component.

## Results

### LukPQ: a new phage encoded leukocidin associated with equids

**Figure 1:**
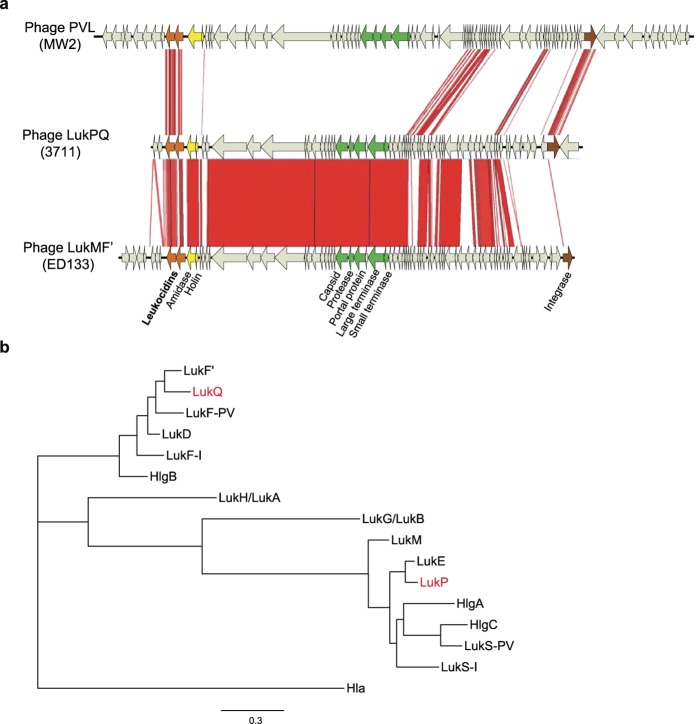
The novel *Staphylococcus aureus* toxin LukPQ in the context of other leukocidins. **(a)** Comparison of the novel prophage ΦSaeq1 in isolate 3711, carrying the equid specific lukPQ, with ΦSaov3 (ruminant strain ED133) and ΦSa2 (human PVL strain MW2). Areas of red show regions conserved between the sequences and homologous coding DNA sequences are marked in the same colour. (**b)** Phylogenetic tree based on amino acid sequences of mature leukocidins, with alpha-hemolysin as an outgroup.

In the genome sequences of a collection of *S. aureus* clonal complex (CC)133 from horses and donkeys we identified a 45 kb prophage (named: ΦSaeq1) that displayed considerable sequence similarity and synteny to the previously reported phage ΦSaov3, which encodes the ruminant LukMF’ (Fig. 1a). ΦSaeq1 was highly conserved among equid CC133 strains and was integrated at a position ~0.5 Mb into the chromosome at approximately the same site as ΦSaov1 and SaPIbov1 in ED133 and RF122, respectively^2^. ΦSaeq1 encoded a novel leukocidin, close to the amidase genes of the phage (Fig. 1a). As the strains carrying this new variant were isolated from two species of *Equidae*, we propose that the new toxin be named LukPQ (P for *Paardachtigen*, Dutch for *Equidae*) and use isolate 3711 as a reference strain for describing this phage and leukocidin locus. Phylogenetic analysis of LukPQ in comparison to the rest of the leukocidin family showed that LukP was most closely related to LukE (91% amino acid identity), whereas LukQ was most similar to the ruminant associated LukF’ (83% amino acid identity), but also shared 80% amino acid sequence with LukD (Fig. 1b). Molecular modelling of LukP and LukQ confirmed that both subunits adopt classical leukocidin folds (Supplementary Fig. 1). To further validate the association with equids we screened our collection of sequenced genomes by BLASTn and found *lukPQ* with 99–100% nucleotide identity in 29 isolates from 5 different clonal complexes (CC1, CC133, CC350, CC522, CC1660), and from a broad geographical distribution of countries (Brazil, Switzerland, Tunisia, United Kingdom), primarily from equid hosts, but also in 5 isolates from ruminants (Supplementary Table 1). In the majority of positive isolates (96%), *lukPQ* was present on a phage, but in two strains from Brazilian buffalo, *lukPQ* was flanked by only two phage-related genes (amidase and holin); the remainder of the phage was not present in the genome of these strains. Between CCs, the phage encoding *lukPQ* showed considerable variation, but *lukPQ* was highly conserved, showing only few SNPs, which were associated with clonal lineage (Supplementary Table 1), comparable to what has been shown for *pvl*-encoding phages^16^.

We estimated the prevalence of *lukPQ* in an international collection of equid *S. aureus* isolates (The Netherlands (unpublished), Austria^17^, the United States^18^, Sweden^19^, Portugal^20^, Italy^21^ and Spain^22^) using a PCR assay to identify the three prophage-encoded leukocidins (*lukSF-PV, lukMF’* and *lukPQ*). *lukPQ* was present in 29 out of 194 strains (15%, 95% CI: 10 to 21%) from the Netherlands, Italy and Portugal, whereas *lukSF-PV* and *lukMF’* were only found once and twice, respectively (Supplementary Table 2). Between isolate collections, the prevalence of *lukPQ* differed considerably. In the Dutch collection LukPQ was found in 25 of 74 isolates (34%); interestingly this included 11 out of 21 isolates (52%) from the *spa*-type t011 (CC398) - a lineage that has been reported to be specifically associated with horses^23^.

### LukPQ preferentially kills horse neutrophils

**Figure 2:**
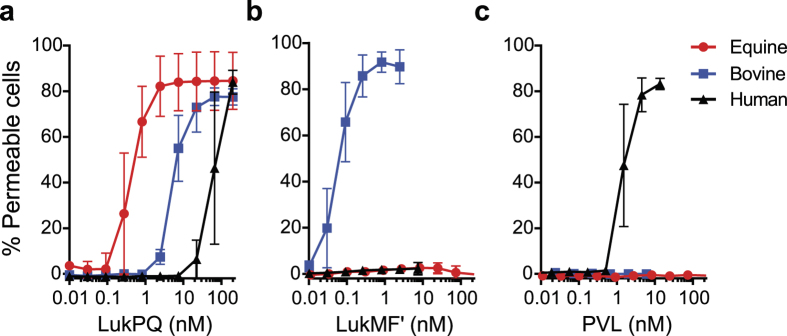
LukPQ is a potent killer of equine neutrophils. (**a**,**b** and **c)** Equine, bovine and human neutrophils were analysed for pore formation upon incubation with LukPQ (A), LukMF’ (B), and LukSF-PV (C). Mean percentages of permeable cells ± standard deviation (SD) are shown (n = 3–5).

As there was evidence for the association of LukPQ with equid hosts, we sought to identify if it exhibited specific activity against horse neutrophils, leukocytes known to be instrumental in the host defence against *S. aureus*^24^. Equine, bovine and human neutrophils were incubated with the three prophage-encoded leukocidins with an assumed host specificity (LukPQ (putatively equid), LukMF’ (ruminant) and LukSF-PV (human)) and pore formation was quantified in a dose dependent manner. Equine neutrophils were highly susceptible to LukPQ-induced lysis with a half-maximal lytic concentration (EC_50_) of 0.46 nM (±SD 0.23) (Fig. 2a). This was higher than the EC_50_ of LukMF’ on bovine neutrophils (0.08 nM (±SD 0.02), p < 0.001) (Fig. 2b)^14^, but significantly lower than the EC_50_ of LukSF-PV on human neutrophils (1.63 nM (±SD 0.66), p = 0.006) (Fig. 2c). Both LukMF’ and LukSF-PV were unable to induce pore formation in equine neutrophils, emphasizing their described host restrictions^13^,^15^. LukPQ, however, was able to permeabilise both human and bovine neutrophils, but at significantly higher EC_50_’s (45.82 nM (±SD 11.10) and 5.68 nM (±SD 1.64) respectively, both p < 0.0001) (Fig. 2a).

### LukPQ acts on CXCRA and CXCR2

**Figure 3:**
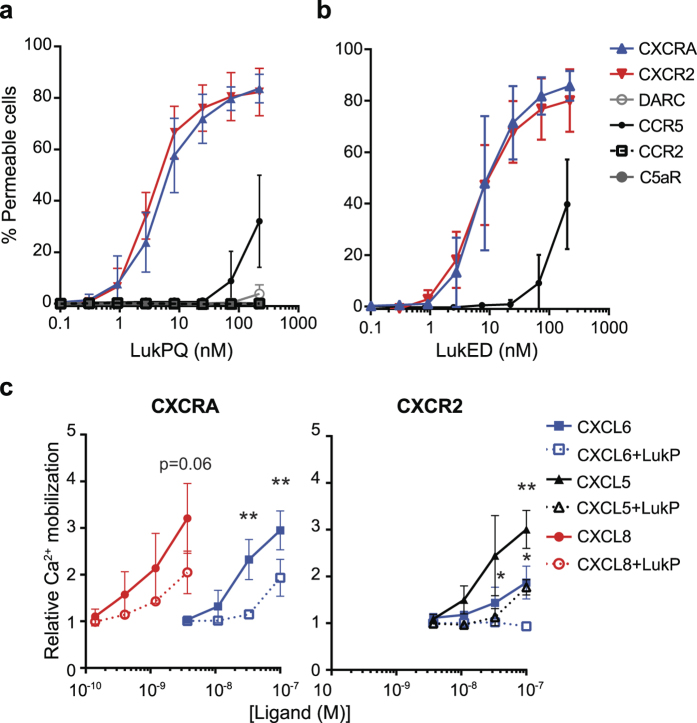
CXCRA and CXCR2 mediate pore formation by LukPQ in equine neutrophils. (**a)** Pore formation in HEK293T cells stably transfected with equine CCR2, CCR5, C5aR, CXCRA, CXCR2 and the Duffy antigen receptor (DARC) upon treatment with LukPQ. Mean percentages of permeable cells ± SD are shown (n = 3). (**b)** HEK293T cells stably transfected with equine CXCRA, CXCR2 and CCR5 were incubated with LukED and analysed for pore formation. Mean percentages of permeable cells ± SD are shown (n = 3). (**c)** Relative calcium mobilization by CXCRA and CXCR2 transfected HEK293T cells preincubated with buffer or 10 μg/ml LukP upon stimulation with CXCL5, CXCL6 and CXCL8. Bars indicate SD with n = 3. Statistically significant effects of preincubation with LukP are indicated. **P < 0.01 and *P < 0.05. Pre-incubation with LukP resulted in a significant decrease in calcium mobilization in both CXCRA and CXCR2 cells stimulated with CXCL6 (p < 0.01 and p < 0.05 respectively), and in CXCR2 cells stimulated with CXCL5 (p < 0.05). A trend was seen in CXCRA cells stimulated with CXCL8 (p = 0.06).

Based on the high degree of similarity (91% amino-acid identity) between receptor binding components LukE and LukP, we hypothesized that the most likely receptors for LukPQ would comprise CXCR1, CXCR2, CCR5, and the Duffy antigen receptor (DARC) analogous to LukED^10^,^11^. We cloned and expressed the equine homologues of these receptors (the putative CXCR1 homologue in equids is CXCRA^25^) and CCR2 and C5aR in HEK293T cells and exposed these cells to LukPQ and LukED. This identified CXCRA and CXCR2 as the major receptors for LukPQ with EC_50_’s of 5.81 nM (±SD 3.9) and 3.46 nM (±SD 1.09) respectively (Fig. 3a). Pore formation through CCR5 was less efficient (EC_50_ > 270 nM), while no pore formation was observed in HEK293T cells expressing DARC, C5aR or CCR2. Additionally, we showed that LukED also permeabilises cells carrying equine CXCRA, CXCR2 and CCR5 at efficiencies similar to LukPQ (EC_50_’s of 8.97 nM (±SD 5.74) for CXCRA and 6.93 nM (±SD 3.24) for CXCR2) (Fig. 3b). To further investigate the interaction of the S-component LukP with the CXCRA and CXCR2 receptors, we tested its ability to functionally antagonize stimulation of these receptors. The horse CXCRA and CXCR2 receptors expressed on HEK293T cells were shown to respond to stimulation with human CXCL6 and CXCL8 (ligands for CXCRA) or CXCL5 and CXCL6 (ligands for CXCR2) by intracellular calcium mobilization (Fig. 3c). After priming the CXCRA and CXCR2 transfected cells with LukP, we observed that intracellular calcium mobilization upon stimulation with their specific cytokines was significantly reduced. This suggests that LukP interacts with CXCRA and CXCR2 at the ligand-binding site of these receptors and has immunomodulatory properties when present as a single component. Alternatively, it may be that LukP induces internalization of the receptor, resulting in less surface receptor and therefore in reduced calcium mobilization.

### LukPQ and LukED exhibit different species specificities

**Figure 4:**
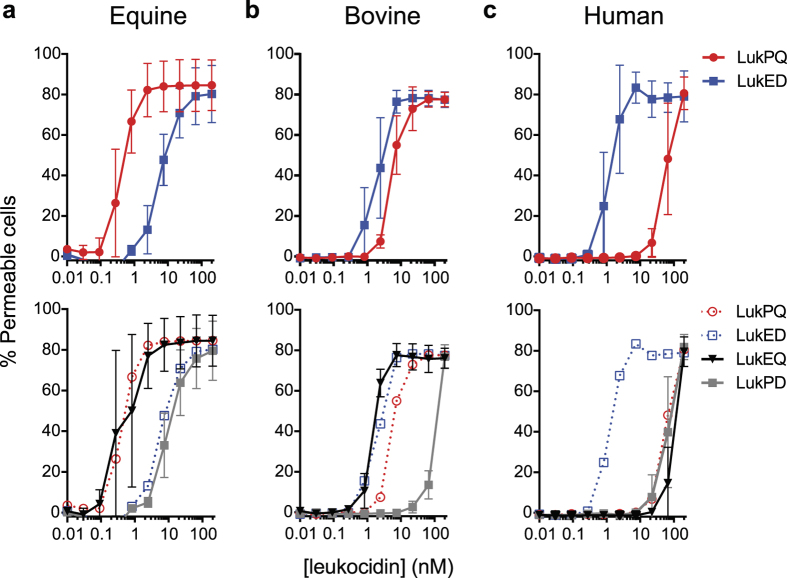
LukPQ and LukED exhibit distinct species specificities in an F-component-dependent manner. Pore formation in equine (**a**), bovine (**b**) and human (**c**) neutrophils upon incubation with LukPQ, LukED, LukEQ or LukPD. Mean percentages of permeable cells ± SD are shown (n = 3–5).

While the presence of *lukPQ* was enriched in equid isolates, the closely related *lukED*, located on a pathogenicity island, is present in most *S. aureus* isolates^5^. We identified that all of the sequenced equid strains in our collection (Supplementary Table 1) that harboured *lukPQ* also harboured *lukED*, although in CC133 strains, the *lukE* gene was disrupted by a nonsense mutation in amino acid position 174, as has been reported for other CC133 strains^2^. In order to assess the additional value of LukPQ in equid isolates in comparison to the ubiquitously present LukED, we compared the cytotoxicity of both toxins on equine, bovine and human neutrophils. Interestingly, when comparing EC_50_ values, LukED is a significantly less potent killer of equine neutrophils than LukPQ with an EC_50_ of 6.62 nM (±SD 4.45) (p = 0.004) (Fig. 4a). This finding was not apparent in the data from the receptors expressed in HEK293T cells, where LukPQ and LukED displayed almost equal toxicity (p = 0.73 for CXCRA and p = 0.46 for CXCR2 expressing cells) (Fig. 3). Human neutrophils are permeabilised significantly more efficiently by LukED than by LukPQ (p < 0.001), while for bovine neutrophils the increased efficiency of LukED is minimal and non-significant (p = 0.079) (Fig. 4b and c).

### The F-component is involved in host-specificity

Next, because of the high degree of similarity between LukP and LukE, we analysed the effects of the non-canonical toxin pairs LukPD and LukEQ on the different neutrophils. LukEQ showed a significant increase in pore formation in equine neutrophils as compared to LukED with an EC_50_ of 0.74 nM (±SD 0.59) (p = 0.007) and was as potent as the native pair LukPQ (p = 0.98) (Fig. 4a). This suggests that LukQ is involved in host specificity to horse neutrophils, a finding that was corroborated by the poor activity of other non-canonical pair: LukPD. Against bovine neutrophils, LukEQ and LukED displayed equal activity with EC_50_’s of 1.51 nM (±SD 0.47) and 2.17 nM (±SD 1.31) respectively (p = 0.9), and the EC_50_ of LukEQ was marginally better than the EC_50_ of LukPQ (5.68 nM (±SD 1.64) p = 0.032, Fig. 4b), suggesting that LukE has a greater specificity for bovine neutrophils than LukP. Finally, against human neutrophils the canonical combination LukED displayed significantly higher activity than all other pairs (p < 0.001), which displayed low-level activity– suggesting that the targeting of human neutrophils requires both LukE and LukD (Fig. 4C). Taken together, the results demonstrate that both the S and F-components of LukED and LukPQ are involved in host specificity and importantly, reveal a previously unrecognised role for a leukocidin F-component in host specificity.

## Discussion

In this study, we describe a new member of the *S. aureus* bicomponent pore-forming toxin family: LukPQ, which is phage-encoded and associated with equid hosts. In accordance with its host distribution, we showed that LukPQ displays an enhanced cytotoxicity towards equine neutrophils. This suggests an important role for LukPQ in the evasion of the host defence mechanism of *S. aureus* in equids, in line with the assumed function of other phage-encoded leukocidins (LukMF’ and PVL) that have a similarly host-specific function^13^,^14^ and distribution^26^ (Supplementary Table 3). *S. aureus* regularly causes problems in equine hospitals, leading primarily to joint, skin and wound infections^27^. Patient-to-patient transmission and outbreaks within equine hospitals as well as zoonotic transmission have been documented^18^,^19^,^28^,^29^,^30^. Recently, an epidemic subclone of CC398 MRSA was shown to have spread within and between equine hospitals^23^. This subclone consisted almost exclusively of spa-type t011 strains, which in our study had a high prevalence of LukPQ. Leukocidins protect *S. aureus* from migrating neutrophils, which are the hosts first line of defence^24^, by creating a protective zone around it^14^, enabling it to reproduce after initial entry into a new host. Likewise, LukPQ may enhance the transmission between equid hosts, driving the success of this clone in equine hospitals. However, further evaluation of the clinical impact of LukPQ in equid infection is required.

**Figure 5:**
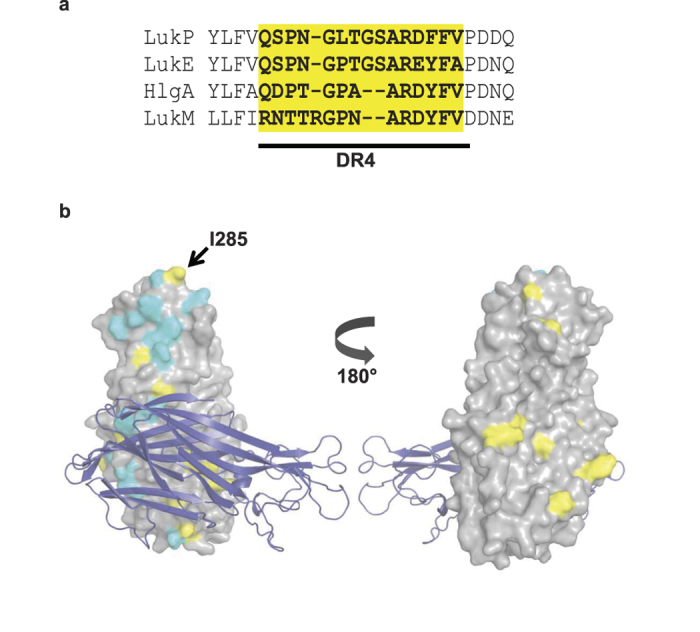
Unique residues in F-components may underlie functional specificity. (**a)** Structure-guided alignment of the DR4 region (highlighted yellow) in the rim domain of LukE, LukP, HlgA and LukM. (**b)** Homology model of the LukPQ heterodimer with LukP as a cartoon and LukQ as a surface representation. Residues unique to LukQ, but identical between LukD and LukF’ are coloured yellow; residues that differ in all three toxins are coloured cyan. The position of isoleucine 285 in the rim domain is annotated.

The γ-hemolysins and LukED target a broad host range and are widely distributed amongst *S. aureus* lineages^15^,^31^,^32^,^33^, consistent with a more generalist function. LukPQ demonstrates host specificity, but has a broader host range than LukMF’ and PVL as at higher concentrations it is capable of lysing bovine and to some extent human neutrophils. We demonstrated that LukPQ targets CXCRA and CXCR2, the equine CXCL8 (IL-8) receptors expressed on neutrophils^34^, as well as CCR5, albeit with lower affinity. While the receptor tropism of LukPQ and LukED is similar, we found a species-dependent difference in cytotoxicity towards neutrophils: LukPQ is more toxic to equine neutrophils than LukED, while the opposite is true for human neutrophils. The S-components LukE and LukP are highly similar. The rim domain, particularly the DR4 region, of the S-component of the toxin is important for receptor binding. Consistent with their shared receptor specificity, the DR4 regions of LukE and LukP are almost identical, whilst that of LukM, which binds CCR1, is considerably different (Fig. 5A). The DR4 region of HlgA, which also binds CXCR1 and CXCR2, is highly similar to that of LukE and LukP, but lacks a GS insertion which may explain why HlgA also targets CCR2 rather than CCR5^12^ (Fig. 5A).

Analysis of the effect of the non-canonical pairs LukPD and LukEQ suggests that the F-components LukD and LukQ (which share only 80% sequence identity) are the key determinants of the difference in activity between LukPQ and LukED against equine and human neutrophils, whereas LukD and LukQ have equal specificity for bovine neutrophils. Comparing LukQ, LukD and LukF’ identifies 20 residues that are unique to LukQ, but which are conserved between LukD and LukF’ and a further 13 residues that differ between all three toxins (Supplementary Fig. 2). Some of these LukQ-unique residues are found in the likely interface for binding with LukP, and one of the unique residues, I285 in the LukQ rim domain, maps to a position previously identified in LukF-PV as important for interaction with the cell membrane^35^ (Fig. 5B). Further studies involving chimeric F-components may yield insight in the actual importance of these residues. Still, the variable residues do not group onto one specific surface, so it is unclear whether the host specificity mediated through LukD and LukQ stems from the interaction between the F-component and the S-component, or from the interaction between the F-component and the cell membrane. Although F-components do not interact with the cognate GPCR receptors of the leukocidins^9^, LukF-PV has been suggested to bind to an F-component receptor prior to complex formation, possibly explaining the differences in activity between canonical and non-canonical combinations of F-components with LukS-PV^36^,^37^. In the case of LukPQ, we found no significant binding of the F-component to equine neutrophils (Supplementary Fig. 3), suggesting that interaction with an F-component receptor prior to pore-formation is unlikely. However, there is a possibility that F-components recruit a different receptor to the complex of alternating S- and F-components during the pore formation process. Involvement of such a receptor might explain the difference in species specificity of LukED and LukPQ. Future studies will be needed to elucidate the molecular mechanism of pore formation and identify all players involved in the process^38^.

In conclusion, we describe a novel leukocidin with a high sequence similarity to LukED, but we show that the small differences in amino-acid sequence of the S-components in combination with a different F-component leads to a substantial change in affinity for neutrophils of various host species, and therefore to host specificity.

## Methods

### Ethics statement

All experiments were performed in accordance with relevant guidelines and regulations. Written informed consent was obtained from all human blood donors in accordance with the Declaration of Helsinki. The medical ethics committee of the University Medical Center Utrecht (The Netherlands) approved the use of human venous blood for this study. The use of blood from cattle was approved by the Ethical Committee for Animal Experiments of the Utrecht University (Permit No. DEC2012.II.10.152) and conducted according to national regulations.

### Bacterial strains and genome sequencing

Strains used in this study were isolated in the course of previous and on-going studies^39^,^40^ or collected as part of routine surveillance. Genomic DNA was extracted with the MasterPure Gram-positive DNA purification kit (Cambio, United Kingdom). HiSeq sequencing was performed according to the manufacturer’s protocol (Illumina, Inc., United States). Phage identification was performed using PHAST^41^. The nucleotide sequence of the LukPQ positive phage from strain 3711 has been deposited in the Sequence Read Archive database in the European Nucleotide Archive (LT671578).

To estimate the prevalence of the three phage encoded leukocidins, previously reported collections of horse isolates^17^,^18^,^20^,^21^,^22^,^42^ and a selection of isolates from an undescribed Dutch collection were screened by PCR (see Supplementary Methods).

### Leukocyte isolation

Bovine blood was collected from the coccygeal vein of healthy Holstein Friesian cows using a sterile blood collection system with EDTA anti-coagulant (BD Vacutainer). Neutrophils were isolated by using Percoll (1.09176 g/l) centrifugation^14^. Human blood was collected in heparin tubes from healthy volunteers and neutrophils were isolated by Ficoll/Histopaque centrifugation^43^. Blood was collected from healthy horses during the slaughter process (and immediately upon death) in tubes containing 3 mM EDTA anticoagulant. Equine neutrophils were isolated using 70 and 85% Percoll gradients as described^44^.

### Cloning, expression and purification of recombinant proteins

Recombinant LukP, LukQ, and LukD proteins were generated in *E. coli* according to methods described previously^45^. See Supplementary Methods for details and primer sequences. Recombinant PVL and LukMF’ used in this study were generated as reported previously^9^,^14^. Recombinant LukE was kindly provided by Thomas Henry (Lyon, France)^46^.

### Cloning of receptor expressing plasmids

Horse genomic DNA was obtained from Zyagen (San Diego, USA). Equine chemokine receptors CXCRA, CXCR2, CCR2, CCR5, C5aR1, and the predicted Duffy antigen receptor (DARC) were amplified from equine genomic DNA by PCR using PfuTurbo DNA polymerase (Stratagene). Primers and accession numbers are listed in Supplementary Table 6. Exons encoding DARC were assembled using overlap extension PCR. All coding sequences were cloned into the pIRESpuro3 vector (Clontech) according to methods described elsewhere^12^. The human Ga16 cDNA (pCISG16 plasmid) was kindly provided by Melvin I. Simon^47^. The Ga16 gene was recloned in between the BstBI and EcoRV sites of the pIREShyg3 vector (Clontech) using the following primers:

5′-AACTATTTCGAAGCCGCCACCATGGCCCGCTCGCTGACCTG-3′ and

5′-ATCGAGGATATCTCACAGCAGGTTGATCTCGTC-3′.

### Cell lines and Transfections

HEK293T cells (a human embryonic kidney cell line obtained from the American Type Culture Collection) were maintained in DMEM supplemented with 10% FCS and 100 U/ml penicillin and 100 μg/ml streptomycin. HEK293T cells were stably transfected with human Gα16 plasmids prior to transfection with equine receptor encoding plasmids. Cells were selected for Gα16 expression using 250 μg/ml hygromycin. Transfections with pIRESpuro3 and pIREShyg3-Gα16 plasmids were performed as described^12^.

### Cell permeability assays

HEK293T cells and neutrophils (3 × 10^6^ cells/ml) were incubated with recombinant LukPQ, PVL, LukED, or LukMF’^14^ in a volume of 50 μl in RPMI, containing 0.05% human serum albumin (Sanquin) for 30 minutes at 37 °C, 5% CO_2_. Cells were analyzed by flow cytometry and pore formation was defined as intracellular staining by 4′,6-diamidino-2-phenylindole (DAPI). Equimolar concentrations of S- and F-components were applied in all assays. For analysis, the percentage of DAPI-positive cells incubated with buffer (spontaneously permeabilised cells) was subtracted from the percentage of DAPI-positive cells that were incubated with toxin. Half maximal lytic concentrations (EC50) were calculated using nonlinear regression analyses in Prism6 (Graphpad Software Inc., USA). EC50 data were log transformed and analysed using one-way ANOVA, followed by Tukey’s multiple comparison test.

### Intracellular Calcium mobilization assays

Calcium mobilization assays with CXCRA and CXCR2 HEK293T cells were performed as described^48^, with slight modifications. Cells were resuspended to 5 × 10^6^ cells/ml in Hanks’ Balanced Salt Solution (HBSS) supplemented with 10 mM HEPES, 0.05% HSA and 25 μM Probenecid and were loaded with 2 μM Fluo-3-AM (Invitrogen) for 1 hour at 37 °C while shaking. Cells were washed, resuspended to 5 × 10^6^ cells/ml in the described HBSS buffer and incubated with buffer or 10 μg/mL LukP for 30 minutes at room temperature. Cells were stimulated with different concentrations of CXCL5, CXCL6, and CXCL8. The increase in calcium mobilization was assessed by flow cytometry for 10 seconds before and up to 70 seconds after addition of the stimulus. Relative calcium mobilization was calculated by dividing the mean fluorescence after stimulation by that of the background. The effect of stimulation with or without LukP was assessed using a general linear model, modelling the interaction between concentration of the ligand and presence or absence of LukP on the relative calcium mobilization.

### Computational analysis and leukocidin homology modelling

Homology models were generated with Modeller (v9.14)^49^. For LukM, LukF’, LukP and LukQ templates were derived either from the Homstraad database or from LukE (PDB ID: 3ROH^50^); whilst human CXCR1 (PDB ID: 2LNL^51^) was used as a template for equine CXCRA. Models were created using thorough MD optimisation and very thorough VTFM optimisation before analysis with the integral DOPE function of Modeller. The model of highest initial quality was further refined and improved using SCWRL4^51^,^52^ and Molprobity^53^. Structural alignments were performed using the superposition function of PYMOL (Schrodinger Inc.). All structural images were generated with PYMOL. Sequence alignments and pairwise identities were determined with Clustal Omega^54^. Topology predictions for the membrane spanning receptor proteins were calculated using the Constrained Consensus Topology prediction server^55^,^56^.

## Additional Information

**How to cite this article**: Koop, G. *et al*. Identification of LukPQ, a novel, equid-adapted leukocidin of *Staphylococcus aureus.* *Sci. Rep.* **7**, 40660; doi: 10.1038/srep40660 (2017).

**Publisher's note:** Springer Nature remains neutral with regard to jurisdictional claims in published maps and institutional affiliations.

## Supplementary Material

Supplementary Information

Supplementary Table 1

Supplementary Table 3
