# Supplementary material for: Identification of LukPQ, a novel, equid-adapted leukocidin of Staphylococcus aureus
Source: Sci Rep. 2017 Jan 20;7:40660. doi: 10.1038/srep40660 (PMC5247767; doi:10.1038/srep40660)
Supplement: Supplementary Information [file srep40660-s1.pdf]

Supplementary Information

Identification of LukPQ, a novel, equid-adapted leukocidin of *Staphylococcus aureus*

Gerrit Koop,[1*†] Manouk Vrieling,[2†] Daniel M. L. Storisteanu,[3] Laurence S. C. Lok,[3] Tom Monie,[4,5] Glenn van Wigcheren[2], Claire Raisen,[5] Xiaoliang Ba,[5] Nicholas Gleadall,[5] Nazreen Hadjirin,[5] Arjen J. Timmerman,[6] Jaap A. Wagenaar,[6,7] Heleen M. Klunder,[1] J. Ross Fitzgerald,[8] Ruth Zadoks,[9,10] Gavin K. Paterson,[11] Carmen Torres,[12] Andrew S. Waller,[13] Anette Loeffler,[14] Igor Loncaric,[15] Armando E. Hoet,[16,17] Karin Bergström,[18] Luisa De Martino,[19] Constança Pomba,[20] Hermínia de Lencastre[21,22], Karim Ben Slama[23,24], Haythem Gharsa[23], Emily J. Richardson,[25] Edwin R. Chilvers,[3] Carla de Haas,[2] Kok van Kessel,[2] Jos A. G. van Strijp,[2] Ewan M. Harrison,[26‡] Mark A. Holmes[5‡]

Supplementary figures

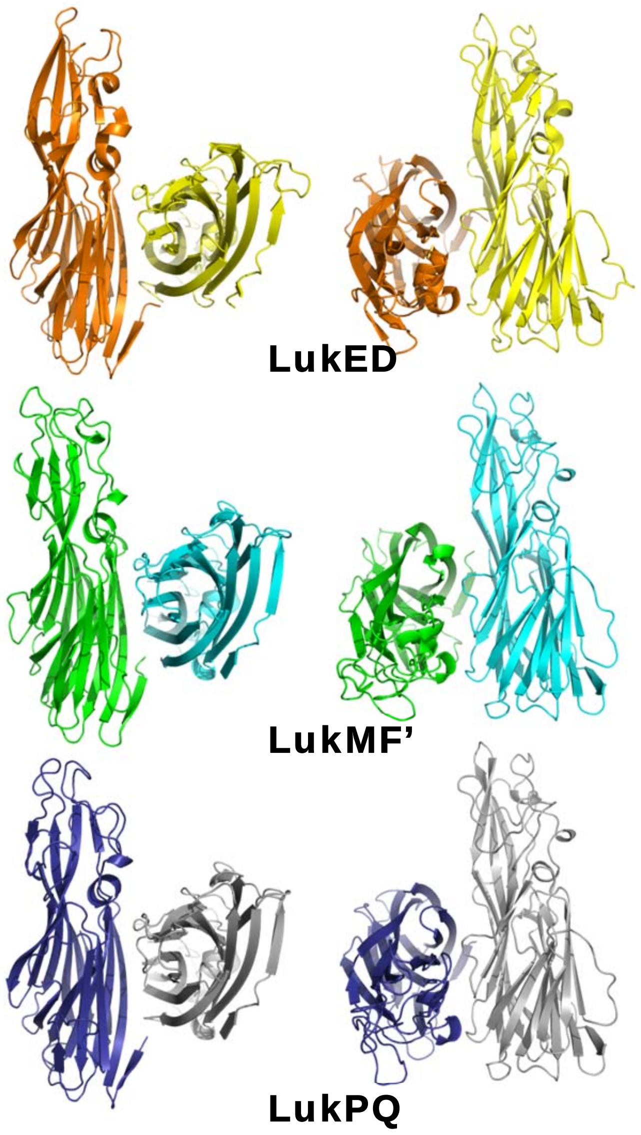

Supplementary Figure 1 | LukPQ and LukMF' are predicted to adopt classical leukocidin structures.

Cartoon representation of the heterodimeric structures of 2-component leukocidin toxins. Top panel – LukED (LukE – orange, LukD – yellow); middle panel LuKMF' (LukM – green; LukF' – Cyan); bottom panel LukPQ (LukP – blue, LukQ – silver). LukE and LukD structures are derived from the PDB co-ordinates 3ROH and 4Q7G respectively, LukM, LukF', LukP and LukQ are homology models. Heterodimeric complexes were generated by superposition with the structures of HlgA (LukE, LukM, LukP) and HlgB (LukD, LukF', LukQ) obtained from PDB co-ordinates 2QK7.

```
LukD  MKMKKLVKSSVASSIALLLLSNTVDAAQHITPVSEKKVDDKITLYKTTATSDNDKLNISQ
LukF' MKFKNIVKSSVATSITLIMLSNTVDAAQHITPVSEKKVDDKITLYKTTATSDSDKLKISQ
LukQ  MKVKKIVKSSIATSIALIMLSNTVDAAQYITPVNEKKVDDKITLYKTTATSDNDKLNMSQ
      **.*::****:*:**:*::*********:****.******************.***::**
                                          Y
LukD  ILTFNFIKDKSYDKDTLVLKAAGNINSGYKKPNPKDYNYSQFYWGGKYNVSVSSESNDAV
LukF' ILTFNFIKDKSYDKDTLILKAAGNIYSGYTQPTSDSSINSQFYWGAKYNVFVSSESKDSV
LukQ  ILTFNFVKDKSYDKDTLVLKASGNINSGYTKPTSDNSISSQFYWGAKYNVFISSEGNDSV
      ******:**********:***:*** ***.:*. ..   ******.**** :***.:*:*

LukD  NVVDYAPKNQNEEFQVQQTLGYSYGGDINISNGLSGGLNGSKSFSETINYKQESYRTTID
LukF' NIVDYAPKNQNEEFQVQQTLGYSYGGDINIINGLTGGLNGSKSFSETINYKQESYRTTID
LukQ  NVVDYAPKNQNEEFQVQQTLGYSYGGDINISNGLSGKLNGSESFSETISYKQESYRTTID
      *:**************************** ***:* ****:******.***********
                           W                    R
LukD  RKTNHKSIGWGVEAHKIMNNGWGPYGRDSYDPTYGNELFLGGRQSSSNAGQNFLPTHQMP
LukF' RKTNHKSIGWGVEAHKIMNNGWGPYGRDSSDSLYGNELFLGGRQSSSNANQNFLPTHQMP
LukQ  RKTDYKTIGWGVEAHKIMNNGWGPYGRDSYDSIYGNELFLGSRQSSSNANQNFLSTHQMP
      ***::*:********************** *  ********.******* **** *****
                                                 W  FY
LukD  LLARGNFNPEFISVLSHKQNDTKKSKIKVTYQREMDRYTNQWNRLHWVGNNYKNQNTVTF
LukF' ILARGNFNPEFISVLSHKQKDVKKSKIKVTYQREMDRYENFWNNLHWIGYNIKNQKRATH
LukQ  ILARGNFNPEFIGVFSHKQNKNKKSKIKVTYQREMDEYVNYWNGIHWIGFNHKAQNIATH
      :***********.*:****:. **************.* * ** :**:* * * *: .*.

LukD  TSTYEVDWQNHTVKLIGTDSKETNPGV
LukF' TSIYEIDWEKHTVKLVASQSSE-----
LukQ  TSIYEIDWEKNTVKLIDKQAYEKVPS-
     ** **:**::.****: .:: *
```

Supplementary Figure 2 | Multiple sequence alignment of LukD, LukF’ and LukQ.

Clustal Omega was used to generate the alignment and consensus sequence (*= conserved, : = highly conservative substitution, . = weakly conservative substitution). Residues unique to LukQ are highlighted yellow, residues that differ in all three sequences are highlighted cyan in the LukQ sequence. Residues identified as important for phospholipid interaction and pore formation are shown in red, bold and underlined text. The original residues from LukF’ are shown above the alignment in red.

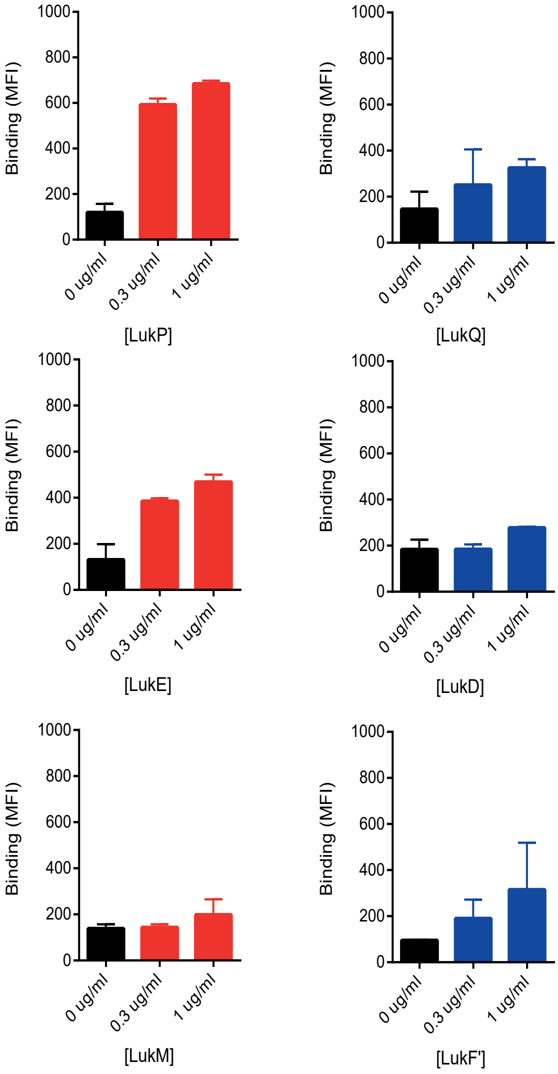

Supplementary Figure 3 | Binding of leukocidin S- and F- components to equine neutrophils.

The mean fluorescence index (MFI) was significantly higher at 0.3 and 1 µg/ml compared to the background (0 µg/ml) for LukP ($P<0.001$) and LukE ($P\leq0.01$). LukD gave slightly higher MFI at 1 µg/ml compared to 0 µg/ml ($P=0.04$), but for all other leukocidins no significant effect of concentration on MFI was found, suggesting no significant binding of these leukocidins to equine neutrophils.

Supplementary tables

Supplementary Table 1 | LukPQ is present in a variety of *Staphylococcus aureus* strains in our collection of sequenced genomes, representing multiple clonal complexes (CC) and countries. Location of the genes, % identity to the reference and single nucleotide polymorphisms (SNPs) present in *lukP* and *lukQ* are reported, as well as presence of other leukocidins.

(See supplementary file 'Supplementary table 1.xlsx')

Supplementary Table 2 | Prevalence of *lukPQ*, *lukMF'*, and *lukSF-PV* in *S. aureus* isolates from horses in 7 countries.

Country	N	*lukPQ*	*lukMF'*	*lukSF-PV*
Netherlands	74	0.34	0.00	0.00
Austria	72	0.00	0.00	0.00
USA	26	0.00	0.04	0.04
Sweden	9	0.00	0.00	0.00
Portugal	5	0.40	0.20	0.00
Italy	4	0.50	0.00	0.00
Spain	4	0.00	0.00	0.00
Total	194	0.15	0.01	0.01

Supplementary Table 3 | Systematic review of the literature for prophage-encoded leucocidins (LukSF-PV, LukMF') in *S. aureus* isolated from animals, showing that the prevalence of leucocidins is generally low in strains from host species that are insensitive to that specific leucocidin.

(See supplementary file 'Supplementary table 3.xlsx')

Supplementary Table 4 | Primers sequences used for producing purified recombinant protein of LukP, LukQ and LukD. Restriction enzyme recognition sites are underlined.

Gene	Primer sequence
lukP	5'-CGGGATCCAATACTAATATTGAAAACATTG-3'
	5'-ATATGCGGCCGCTCAATTGTGTCCTTTCACTTTAA-3'
lukQ	5'-CGGGATCCGCTCAATATATTACACCTGTTA-3'
	5'-ATATGCGGCCGCTTATGAAGGAACTTTTTCGTAAG-3'
lukD	5'-CGGGATCCGCTCAACATATCACACCTGTAAG-3'
	5'-ATATGCGGCCGCTTATACTCCAGGATTAGTTTC-3'

Supplementary Table 5 | Primers sequences used for screening *S. aureus* isolates for the presence of the phage-encoded leukocidin genes.

Gene	Primer sequence	Amplicon size (bp)
lukMF'	ATCAATCGGCTGGGGTGTCGAG	125
	TCGAGCTACTCTGTCTGCCACCT	
lukPQ	CCTGATGGTGAACTGTCAGCGCAT	939
	TTGTGTGCCTCGACACCCCAAC	
lukSF-PV	TGGTGATGGCGCTGAGGTAGTCA	360
	CCATTACCTCCTGTTGATGGACCAC	

Supplementary Table 6 | Primer sequences used to generate equine receptor expressing plasmids. Restriction enzyme recognition sites are underlined.

Gene	Accession number	Primer sequence
CXCRA	*XM_001491012.4*	5'-CGGAATTCATGACTATCATCCTGCAAGATG-3'<br>5'-ATATGCGGCCGCCTAGAGCGTGATAGAAGTGTTC-3'
CXCR2	*XM_005610605.1*	5'-CGGAATTCATGGGAGAATTTAACTTTGC-3'<br>5'-ATATGCGGCCGCCTAGAGCGTGATAGAAGTGTTC-3'
CCR2	*NM_001097606.2*	5'-CGGAATTCATGGATGGCAACAACACATTTC-3'<br>5'-ATATGCGGCCGCTTACAAACCAGCTGAGACTTC-3'
CCR5	*NM_001091534.1*	5'-CGGAATTCATGGATTATCAGACGACAAG-3'<br>5'-ATATGCGGCCGCTCACAAGCCAACAGAGATTTC-3'
C5aR	*XM_001503145.3*	5'-CGGAATTCATGGCCTCCATGGACAATAC-3'<br>5'-ATATGCGGCCGCTCACACGGCCTGGCACTTCTG-3'
DARC Part A (N-term)	*XM_001490641.3*	5'-CGGAATTCATGGCACTTATCTTGGAACCAC-3'<br>5'-GCACCAGGTGGACACCCCCGTGCATGCAGTTCCCCAT-3'
DARC Part B (C-term)	*XM_001490641.3*	5'-ACTGCATGCACGGGGGTGTCCACCTGGTGCCTGAG-3'<br>5'-ATATGCGGCCGCTTAATTCAGCTTGACAGGTG-3'

Supplementary Methods

Cloning, expression and purification of recombinant proteins

Recombinant LukP, LukQ, and LukD proteins were generated in *E. coli* according to methods described previously[76]. The coding sequences of LukP and LukQ were amplified from genomic DNA of *S. aureus* 3711 and LukD was amplified from genomic DNA of *S. aureus* USA300 using the primers given in Supplementary Table 4. Coding sequences were amplified by PCR with Phusion High-Fidelity Polymerase (Thermofisher). Subsequently, LukP and LukQ were cloned into the pRSETB vector (Invitrogen), which was modified to encode proteins with a non-cleavage N-terminal 6xHIS tag. Protein expression was performed in *E. coli* Rosetta Gami (DE3)pLysS and induced with 1 mM Isopropyl β-D-1-iogalactopyranoside (IPTG). Recombinant proteins were isolated from a HiTrap chelating HP column under native conditions and eluted using an imidazole gradient. Finally, proteins were stored in PBS and confirmed to be highly pure (>95%) using SDS-electrophoresis.

Binding assays

Equine neutrophils (3 x 10^6 cells/mL) were incubated with different concentrations of the polyhistidine-tagged leukocidins for 30 minutes on ice in a total volume of 50 µl in RPMI containing 0.05% human serum albumin (Sanquin). Cells were subsequently washed and incubated with a fluorescein isothiocyanate (FITC)-conjugated mouse anti-his antibody (Life Span Biosciences). After 30 min of incubation on ice, cells were washed twice and analyzed by flow cytometry. Leukocidin binding was expressed by the Mean fluorescence intensities (MFI) of the analysed cells. For each leukocidin, MFI at 0.3 and 1 ug/ml was compared to the background level (0 ug/ml) using a general linear model to assess whether significant binding occurred.

Screening of additional *S. aureus* collections and the literature to estimate the prevalence of phage-encoded leukocidins

Authors from publications that describe *S. aureus* strains cultured from horses were contacted by email and asked for permission to screen their strain collection with PCR. A total of 7 strain collections from 7 different countries were screened, comprising a total of 194 strains. The strains were tested by PCR using the primers reported in Supplementary Table 5. From the Dutch horse-strain collection, 74 selected isolates were screened and associations between presence or absence of the *lukPQ* genes and spa-type, presence of the *mecA* gene, and clinical presentation (purulent or not) were tested by Chi-squared tests or Fishers exact test.

To assess the prevalence of LukMF’ and PVL in isolates described in the literature, we searched in Scopus (www.scopus.com) using the following key words: aureus AND [panton-valentine leucocidin OR pvl OR leucocidin OR luk-pv OR luks-pv OR lukf-pv OR lukmf OR lukm]. From articles describing *S. aureus* cultured from animals or food published before 16 January 2015, the total number of isolates tested and the number of isolates positive for

either leukocidin was extracted and the prevalence of LukMF’ and PVL positive isolates was calculated.

Supplementary references

1. Sieber, S. *et al*. Evolution of multidrug-resistant *Staphylococcus aureus* infections in horses and colonized personnel in an equine clinic between 2005 and 2010. *Microb. Drug Resist.* **17**, 471-478 (2011).

2. Aires-de-Sousa, M. *et al*. Characterization of *Staphylococcus aureus* isolates from buffalo, bovine, ovine, and caprine milk samples collected in Rio de Janeiro State, Brazil. *Appl. Environ. Microbiol.* **73**, 3845-3849 (2007).

3. Gharsa, H. *et al*. High diversity of genetic lineages and virulence genes in nasal *Staphylococcus aureus* isolates from donkeys destined to food consumption in Tunisia with predominance of the ruminant associated CC133 lineage. *BMC Vet. Res.* **8** (2012).

4. Gharsa, H. *et al*. Molecular characterization of *Staphylococcus aureus* from nasal samples of healthy farm animals and pets in Tunisia. *Vector-Borne Zoonotic Dis.* **15**, 109-115 (2015).

5. Monecke, S. *et al*. Microarray-based genotyping of *Staphylococcus aureus* isolates from camels. *Vet. Microbiol.* **150**, 309-314 (2011).

6. Yamada, T. *et al*. Leukotoxin family genes in *Staphylococcus aureus* isolated from domestic animals and prevalence of lukM-lukF-PV genes by bacteriophages in bovine isolates. *Vet. Microbiol.* **110**, 97-103 (2005).

7. Schlotter, K. *et al*. Leukocidin genes lukF-P83 and lukM are associated with *Staphylococcus aureus* clonal complexes 151, 479 and 133 isolated from bovine udder infections in Thuringia, Germany. *Vet. Res.* **43** (2012).

8. Moser, A., Stephan, R., Corti, S. & Johler, S. Comparison of genomic and antimicrobial resistance features of latex agglutination test-positive and latex agglutination test-negative *Staphylococcus aureus* isolates causing bovine mastitis. *J. Dairy Sci.* **96**, 329-334 (2013).

9. Monecke, S., Kuhnert, P., Hotzel, H., Slickers, P. & Ehricht, R. Microarray based study on virulence-associated genes and resistance determinants of *Staphylococcus aureus* isolates from cattle. *Vet. Microbiol.* **125**, 128-140 (2007).
10. Fueyo, J. M. *et al*. Cytotoxin and pyrogenic toxin superantigen gene profiles of *Staphylococcus aureus* associated with subclinical mastitis in dairy cows and relationships with macrorestriction genomic profiles. *J. Clin. Microbiol.* **43**, 1278-1284 (2005).
11. Hata, E. *et al*. Genetic variation among *Staphylococcus aureus* strains from bovine milk and their relevance to methicillin-resistant isolates from humans. *J. Clin. Microbiol.* **48**, 2130-2139 (2010).
12. Haveri, M., Roslöf, A., Rantala, L. & Pyörälä, S. Virulence genes of bovine *Staphylococcus aureus* from persistent and nonpersistent intramammary infections with different clinical characteristics. *J. Appl. Microbiol.* **103**, 993-1000 (2007).
13. Vautor, E. *et al*. Genetic differences among *Staphylococcus aureus* isolates from dairy ruminant species: A single-dye DNA microarray approach. *Vet. Microbiol.* **133**, 105-114 (2009).
14. Haveri, M., Hovinen, M., Roslöf, A. & Pyörälä, S. Molecular types and genetic profiles of *Staphylococcus aureus* strains isolated from bovine intramammary infections and extramammary sites. *J. Clin. Microbiol.* **46**, 3728-3735 (2008).
15. Chu, C. *et al*. Differences in virulence genes and genome patterns of mastitis-associated *Staphylococcus aureus* among goat, cow, and human isolates in Taiwan. *Foodborne Pathog. Dis.* **10**, 256-262 (2013).
16. Wedley, A. L. *et al*. Carriage of *Staphylococcus* species in the veterinary visiting dog population in mainland UK: Molecular characterisation of resistance and virulence. *Vet. Microbiol.* **170**, 81-88 (2014).
17. Monecke, S. *et al*. Detection of mecC-Positive *Staphylococcus aureus* (CC130-MRSA-XI) in Diseased European Hedgehogs (Erinaceus europaeus) in Sweden. *PLoS ONE* **8** (2013).

18. Huijsdens, X. W. *et al*. Community-acquired MRSA and pig-farming. *Ann. Clin. Microbiol. Antimicrob.* **5** (2006).

19. van Duijkeren, E. *et al*. Transmission of methicillin-resistant *Staphylococcus aureus* strains between different kinds of pig farms. *Vet. Microbiol.* **126**, 383-389 (2008).

20. Gharsa, H. *et al*. Prevalence, antibiotic resistance, virulence traits and genetic lineages of *Staphylococcus aureus* in healthy sheep in Tunisia. *Vet. Microbiol.* **156**, 367-373 (2012).

21. De Santis, E., Mureddu, A., Mazzette, R., Scarano, C. & Bes, M. Detection of enterotoxins and virulence genes in *Staphylococcus aureus* strains isolated from sheep with subclinical mastitis. In: *Mastitis in Dairy Production: Current Knowledge and Future Solutions*, 504-510 (2005).

22. Almeida, L. M., Almeida, M. Z. P. R. B., Mendonça, C. L. & Mamizuka, E. M. Comparative analysis of agr groups and virulence genes among subclinical and clinical mastitis *Staphylococcus aureus* isolates from sheep flocks of the Northeast of Brazil. *Brazilian J. Microbiol.* **44**, 493-498 (2013).

23. Simpson, V. R., Hargreaves, J., Butler, H. M., Davison, N. J. & Everest, D. J. Causes of mortality and pathological lesions observed post-mortem in red squirrels (*Sciurus vulgaris*) in Great Britain. *BMC Vet. Res.* **9** (2013).

24. Simpson, V. R. *et al*. Association of a lukM-positive clone of *Staphylococcus aureus* with fatal exudative dermatitis in red squirrels (*Sciurus vulgaris*). *Vet. Microbiol.* **162**, 987-991 (2013).

25. Walther, B. *et al*. Methicillin-resistant *Staphylococcus aureus* (MRSA) isolated from small and exotic animals at a university hospital during routine microbiological examinations. *Vet. Microbiol.* **127**, 171-178 (2008).

26. Loncaric, I. *et al*. Identification and characterization of methicillin-resistant *Staphylococcus aureus (*MRSA) from Austrian companion animals and horses. *Vet. Microbiol.* **168**, 381-387 (2014).

27. Weese, J. S. *et al*. Suspected transmission of methicillin-resistant *Staphylococcus aureus* between domestic pets and humans in veterinary clinics and in the household. *Vet. Microbiol.* **115**, 148-155 (2006).

28. Strommenger, B. *et al*. Molecular characterization of methicillin-resistant *Staphylococcus aureus* strains from pet animals and their relationship to human isolates. *J. Antimicrob. Chemother.* **57**, 461-465 (2006).

29. Wendlandt, S. *et al*. Resistance phenotypes and genotypes of methicillin-resistant: *Staphylococcus aureus* isolates from broiler chickens at slaughter and abattoir workers. *J. Antimicrob. Chemother.* **68**, 2458-2463 (2013).

30. Kwon, N. H. *et al*. Staphylococcal cassette chromosome mec (SCCmec) characterization and molecular analysis for methicillin-resistant *Staphylococcus aureus* and novel SCCmec subtype IVg isolated from bovine milk in Korea. *J. Antimicrob. Chemother.* **56**, 624-632 (2005).

31. Wang, X. *et al*. Antimicrobial susceptibility testing and genotypic characterization of *Staphylococcus aureus* from food and food animals. *Foodborne Pathog. Dis.* **9**, 95-101 (2012).

32. Zecconi, A., Cesaris, L., Liandris, E., Daprà, V. & Piccinini, R. Role of several *Staphylococcus aureus* virulence factors on the inflammatory response in bovine mammary gland. *Microb. Pathog.* **40**, 177-183 (2006).

33. Wang, X. *et al*. Antimicrobial resistance and toxin gene profiles of *Staphylococcus aureus* strains from Holstein milk. *Lett. Appl. Microbiol.* **58**, 527-534 (2014).

34. Benhamed, N. & Kihal, M. Etiology, antimicrobial susceptibility of udder pathogens phenotypic and genotypic characterization of *Staphylococcus aureus* involved in bovine mastitis in Algeria. *Res. J. Appl. Sci.* **8**, 262-267 (2013).

35. Pajić, M. J. *et al*. The prevalence of methicillin resistance and Panton-Valentine leukocidin synthesis genes in *Staphylococcus aureus* isolates of bovine and human origin. *Veterinarski Arhiv* **84**, 205-214 (2014).

36. Bardiau, M. *et al*. Genotypic and phenotypic characterization of methicillin-resistant *Staphylococcus aureus* (MRSA) isolated from milk of bovine mastitis. *Lett. Appl. Microbiol.* **57**, 181-186 (2013).

37. Pu, W. *et al*. High incidence of oxacillin-susceptible mecA-positive *Staphylococcus aureus* (OS-MRSA) associated with bovine mastitis in China. *PLoS ONE* **9** (2014).

38. Zora, Š, Sylva, K. & Renáta, K. Findings of methicillin-Resistant strains of *Staphylococcus aureus* in livestock. *Czech J. Food Sci.* **27**, S236-S241 (2009).

39. Feßler, A. *et al*. Characterization of methicillin-resistant *Staphylococcus aureus* ST398 from cases of bovine mastitis. *J. Antimicrob. Chemother.* **65**, 619-625 (2010).

40. Prashanth, K., Rao, K. R., Reddy, V. P., Saranathan, R. & Makki, A. R. Genotypic characterization of *Staphylococcus aureus* obtained from humans and bovine mastitis samples in India. *J. Global Inf. Dis.* **3**, 115-122 (2011).

41. Lim, S. -. *et al*. Transmission and persistence of methicillin-resistant *Staphylococcus aureus* in milk, environment, and workers in dairy cattle farms. *Foodborne Pathog. Dis.* **10**, 731-736 (2013).

42. van Duijkeren, E. *et al*. Prevalence of methicillin-resistant *Staphylococcus aureus* carrying mecA or mecC in dairy cattle. *Vet. Microbiol.* **171**, 364-367 (2014).

43. Tavakol, M. *et al*. Bovine-associated MRSA ST398 in the Netherlands. *Acta Vet. Scand.*, 28 (2012).

44. Erdem, Z. & Türkyilmaz, S. Molecular typing of methicillin resistant *Staphylococcus aureus* strains isolated from cows and farm workers. *Kafkas Univ. Vet. Fak. Dergisi* **19**, 963-968 (2013).

45. Türkyilmaz, S., Tekbiyik, S., Oryasin, E. & Bozdogan, B. Molecular epidemiology and antimicrobial resistance mechanisms of methicillin-resistant *Staphylococcus aureus* isolated from bovine milk. *Zoonoses Public Health* **57**, 197-203 (2010).

46. Van Duijkeren, E., Wolfhagen, M. J. H. M., Heck, M. E. O. C. & Wannet, W. J. B. Transmission of a Panton-Valentine leucocidin-positive, methicillin-resistant *Staphylococcus aureus* strain between humans and a dog. *J. Clin. Microbiol.* **43**, 6209-6211 (2005).

47. Vanderhaeghen, W. *et al*. Screening for methicillin-resistant staphylococci in dogs admitted to a veterinary teaching hospital. *Res. Vet. Sci.* **93**, 133-136 (2012).

48. Rubin, J. E. & Chirino-Trejo, M. Antimicrobial susceptibility of canine and human *Staphylococcus aureus* collected in Saskatoon, Canada. *Zoonoses Public Health* **58**, 454-462 (2011).

49. Boost, M. V., O'Donoghue, M. M. & Siu, K. H. G. Characterisation of methicillin-resistant *Staphylococcus aureus* isolates from dogs and their owners. *Clin. Microbiol. Inf.* **13**, 731-733 (2007).

50. Walther, B. *et al*. *Staphylococcus aureus* und MRSA-Kolonisierungsraten bei Personal und Hunden in einer Kleintierklinik und deren Assoziation mit dem Auftreten nosokomialer Infektionen. *Berl. Munch. Tierarztl. Wochenschr.* **122**, 178-185 (2009).

51. Corrente, M. *et al*. Characterisation of a catalase-negative methicillin-resistant *Staphylococcus aureus* isolate from a dog. *Vet. Microbiol.* **167**, 734-736 (2013).

52. Grönlund Andersson, U. *et al*. Outbreaks of methicillin-resistant *Staphylococcus aureus* among staff and dogs in Swedish small animal hospitals. *Scand. J. Infect. Dis.* **46**, 310-314 (2014).

53. Davis, J. A. *et al*. Carriage of methicillin-resistant staphylococci by healthy companion animals in the US. *Lett. Appl. Microbiol.* **59**, 1-8 (2014).

54. Stastkova, Z., Karpiskova, S. & Karpiskova, R. Occurrence of methicillin-resistant strains of *Staphylococcus aureus* at a goat breeding farm. *Vet. Med.* **54**, 419-426 (2009).

55. Loncaric, I. *et al*. Characterization of methicillin-resistant *Staphylococcus* spp. carrying the mecC gene, isolated from wildlife. *J. Antimicrob. Chemother.* **68**, 2222-2225 (2013).

56. Walther, B. *et al*. Comparative molecular analysis substantiates zoonotic potential of equine methicillin-resistant *Staphylococcus aureus*. *J. Clin. Microbiol.* **47**, 704-710 (2009).

57. Schwaber, M. J. *et al*. Clonal transmission of a rare methicillin-resistant *Staphylococcus aureus* genotype between horses and staff at a veterinary teaching hospital. *Vet. Microbiol.* **162**, 907-911 (2013).

58. Oliveira, P. S. *et al*. Isolation, pathogenicity and disinfection of *Staphylococcus aureus* carried by insects in two public hospitals of Vitória da Conquista, Bahia, Brazil. *Brazilian J. Inf. Dis.* **18**, 129-136 (2014).
59. Fall, C. *et al*. Epidemiology of *Staphylococcus aureus* in pigs and farmers in the largest farm in Dakar, Senegal. *Foodborne Pathog. Dis.* **9**, 962-965 (2012).
60. Osadebe, L. U., Hanson, B., Smith, T. C. & Heimer, R. Prevalence and characteristics of *Staphylococcus aureus* in Connecticut swine and swine farmers. *Zoonoses Public Health* **60**, 234-243 (2013).
61. Yan, X. *et al*. *Staphylococcus aureus* ST398 from slaughter pigs in northeast China. *International Journal of Medical Microbiology* **304**, 379-383 (2014).
62. Cui, S. *et al*. Isolation and characterization of methicillin-resistant *Staphylococcus aureus* from swine and workers in China. *J. Antimicrob. Chemother.* **64**, 680-683 (2009).
63. Ho, J., O'Donoghue, M., Guardabassi, L., Moodley, A. & Boost, M. Characterization of Methicillin-Resistant *Staphylococcus aureus* Isolates from pig carcasses in Hong Kong. *Zoonoses Public Health* **59**, 416-423 (2012).
64. Kadlec, K. *et al*. Diversity of antimicrobial resistance pheno- and genotypes of methicillin-resistant *Staphylococcus aureus* ST398 from diseased swine. *J. Antimicrob. Chemother.* **64**, 1156-1164 (2009).
65. Franco, A. *et al*. Molecular characterization of spa type t127, sequence type 1 methicillin-resistant *Staphylococcus aureus* from pigs. *J. Antimicrob. Chemother.* **66**, 1231-1235 (2011).
66. Gordoncillo, M. J. *et al*. Detection of methicillin-resistant *Staphylococcus aureus* (MRSA) in backyard pigs and their owners, Michigan, USA. *Zoonoses Public Health* **59**, 212-216 (2012).
67. Fang, H. W., Chiang, P. H. & Huang, Y. C. Livestock-associated methicillin-resistant *Staphylococcus aureus* ST9 in pigs and related personnel in Taiwan. *PLoS ONE* **9** (2014).

68. Lo, Y. P. *et al*. Molecular characterization and clonal genetic diversity of methicillin-resistant *Staphylococcus aureus* of pig origin in Taiwan. *Comp. Immunol. Microbiol. Infect. Dis.* **35**, 513-521 (2012).

69. Vestergaard, M. *et al*. Sccmec type IX element in methicillin resistant *Staphylococcus aureus* spa type t337 (CC9) isolated from pigs and pork in Thailand. *Frontiers Microbiol.* **3** (2012).

70. Smith, T. C. *et al*. Methicillin-resistant *Staphylococcus aureus* (MRSA) strain ST398 is present in midwestern U.S. swine and swine workers. *PLoS ONE* **4** (2009).

71. Velasco, V., Sherwood, J. S., Rojas-García, P. P. & Logue, C. M. Multiplex real-time PCR for detection of *Staphylococcus aureus*, mecA and Panton-Valentine Leukocidin (PVL) genes from selective enrichments from animals and retail meat. *PLoS ONE* **9** (2014).

72. Wardyn, S. E., Kauffman, L. K. & Smith, T. C. Methicillin-resistant *Staphylococcus aureus* in central Iowa wildlife. *J. Wildl. Dis.* **48**, 1069-1073 (2012).

73. Loncaric, I. & Künzel, F. Sequence type 398 meticillin-resistant *Staphylococcus aureus* infection in a pet rabbit. *Vet. Dermatol.* **24**, 370-e84 (2013).

74. Loncaric, I. *et al*. Comparison of ESBL - And AmpC producing enterobacteriaceae and Methicillin-Resistant *Staphylococcus aureus* (MRSA) isolated from migratory and resident population of rooks (Corvus frugilegus) in Austria. *PLoS ONE* **8** (2013).

75. Ünal, N. *et al*. Panton-Valentine leukocidin and some exotoxins of *Staphylococcus aureus* and antimicrobial susceptibility profiles of staphylococci isolated from milks of small ruminants. *Trop. Anim. Health Prod.* **44**, 573-579 (2012).

76. Ko, Y. P. *et al*. Phagocytosis escape by a *Staphylococcus aureus* protein that connects complement and coagulation proteins at the bacterial surface. *PLoS Pathog.* **9**, 1-13 (2013).
